# The Diagnostic and Prognostic Value of Plasmatic Exosome Count in Cancer Patients and in Patients with Other Pathologies

**DOI:** 10.3390/ijms25021049

**Published:** 2024-01-15

**Authors:** Stefano Fais, Mariantonia Logozzi

**Affiliations:** 1 Department of Oncology and Molecular Medicine, Istituto Superiore di Sanità, 00161 Rome, Italy; 2 ExoLab Italia, Tecnopolo d’Abruzzo, 67100 L’Aquila, Italy

The extent of both scientific articles and reviews on extracellular vesicles (EVs) has grown impressively over the last few decades. The publications cover investigations of various kinds of EVs, from human EVs to animal and plant-derived EVs. A high degree of effort has been spent in proposing EVs, mostly those of a nanosized (i.e., exosomes), as a natural source of new disease biomarkers. It is a known fact that both normal and tumor cells release exosomes; after their paracrine release, exosomes are spilled over into the blood, thus circulating through the organism, often ending in organs and compartments far from the site of the original release. The first evidence of this phenomenon is that exosomes may be detected, characterized and quantified in plasma samples of both healthy individuals and tumor patients. Due to their size (30–150 nm), exosomes are invisible particles that may be analyzed through both electron microscopy and other techniques that hijack the peculiar make-up of these nanovesicles. Exosomes express on the surface markers of intracellular vesicles (e.g., endosomes, lysosomes, phagosomes) together with plasma membrane molecules, through which the cellular source of exosomes may be recognized. This peculiarity is due to the various processes of exosome generation, and is particularly due to the multivesicular body (MVB) formation, which is a process of repeated rounds of internal vesicle fusion that involves the plasma membrane as well [1,2,3,4,5]. This process causes exosomes to express an array of molecules (e.g., Tsg101, Alix, CD63, CD9, CD81, HSP-70, Rab5), rendering these nanovesicles phenotypically recognizable. In fact, all the above molecules have been exploited to set up immunocapture-based techniques that have allowed for exosome characterization and quantification [6]. The first clinical study, performed in 148 individuals, was exclusively based on the use of an immunocapture-based ELISA test, through which it was shown that melanoma patients had significantly higher CD63+ plasmatic exosomes compared to healthy individuals, but significantly higher Cav-1 positive exosomes as well, where Cav-1 is considered a surrogate tumor biomarker [7]. However, for the first time, this study supported a new finding that could play a key role in the future clinical management of tumors: melanoma patients present higher exosome levels in their plasma as compared to healthy individuals. In the same study, a preclinical investigation showed that higher plasmatic levels of exosomes correlated with the tumor mass [7]. The in vivo study was also supported by a series of reports showing that the microenvironmental acidity of tumors could exert a key role in determining an increased tumor exosome spill over into the blood stream inasmuch as, in vitro, a low pH condition induces an extensive exosome release by human tumor cells, independently from their histologies [8,9]. The increased low pH-dependent exosome release was consistent with both an increased expression of known tumor biomarkers (e.g., PSA) and a reduced exosome size [8]. In the same study, it was shown that the increased exosome release in acidic conditions correlated to the high plasmatic exosome levels as compared to controls [8]. It appears conceivable that the pH-dependent increase in exosome release may be dependent on one of the functions of the EVs, that is, to scavenge potentially toxic molecules as it has been shown for chemotherapeutics in tumor cells [10] and gold nanoparticles in human normal macrophages [11]. An interesting observation is that both chemical molecules and nanoparticles are released into the exosomes in their native/active form, further supporting the natural ability of exosomes to deliver functional molecules [10,11]. This advantage also includes the ability to deliver functional molecules with a full enzymatic function [12] and a cargo of protons [13]. A fascinating new issue is that exosomes deliver the pathologic prion protein (PrPC) as well [14], which is a glycoprotein anchored to the cell surface by glycosylphosphatidylinositol (GPI). Recent findings have shown the ectopic expression of PrPC in various cancers, including gastric, melanoma, breast, colorectal, pancreatic as well as rare cancers, where PrPC promotes cellular migration and invasion, tumor growth and metastasis [15]. Another topic of interest is that PrPC is delivered by exosomes in a model of prion-infected rodents and PrPC-associated exosomes can be purified in the plasma of the infected animals [15], suggesting that, in general, the identification of PrPC-associated proteins in the plasma of either tumor patients or patients with neurodegenerative diseases may represent a new valuable disease marker.

However, in the last decade, a new technique called nanoparticle tracking analysis (NTA), which is used to determine exosome number and size in samples from both cell culture supernatants and body fluids, has come to support data obtained with immunocapture-based ELISA [16,17]. The NTA analyzes particles ranging from 30 nm to 400–500 nm, thus allowing for the distinguishing of nanovesicles from microvesicles. To date, NTA remains to be considered as the most reliable technique to analyze a mixed population of submicroscopical vesicles in human body fluids. A previous study compared the results obtained from NTA, immunocapture-based ELISA, and nanoscale flow cytometry in exosome preparations obtained from either cell culture supernatants or plasma samples [8]. The results clearly revealed a complete overlapping between the three techniques; however, the immunocapture-based ELISA was incapable of providing information on the EVs’ size, while the nanoscale flow cytometry allowed for the gating of EVs ranging from 100 to 300 nm. Nonetheless, the implementation of these techniques provided valuable information on the number, size, distribution and the phenotyping of EVs from a plasma sample of both tumor patients and either healthy or disease controls. On the basis of these preliminary results, the NTA was performed in plasma samples of patients with prostate cancer and were compared to healthy donors. The results clearly showed that prostate cancer patients had significantly higher exosome levels compared to healthy donors [16], thus strongly supporting the data obtained in the preliminary study [9]. An independent study performed in patients with glioblastoma reported comparable results in displaying higher exosome levels in the plasma of glioblastoma patients [17]. More recently, a longitudinal study performed using the NTA in patients with oral cancers has shown that high plasmatic levels of exosomes may be predictive of a recurrence after surgical treatment [18], supporting a previous investigation which revealed different exosome counts before and after surgical treatment [19].

The importance of these findings is increased by the growing evidence that EVs—particularly exosomes—have not been shown to deliver molecules with a tumor specificity despite being a potential source of disease biomarkers. Glypican-1 is a clear example; in fact, while it has been proposed to be a specific marker of pancreatic cancer, it has displayed a high level of expression in exosome samples obtained in plasma from patients with other cancers [20]. Nonetheless, it may be of some help when performed in combination with well-known tumor biomarkers [21,22]. It has in fact been demonstrated that plasmatic exosomes express high levels of acknowledged tumor markers such as PSA, which distinguishes prostate cancer from both healthy and inflammatory states [23]. Comparable studies should also be performed for other acknowledged tumor markers that have been demonstrated to be delivered by plasmatic exosomes (e.g., CEA) [24]. However, the increased exosome plasmatic levels have a double importance in clinical oncology. In fact, growing scientific evidence supports a key role of exosomes in tumor metastasis, [25,26,27]; on the other hand, the involvement in tumor metastasis increases the importance of exosome count in the plasma of tumor patients in further refining a prognostic evaluation. The circulating mass of tumor exosomes may represent a real danger for the patients’ body with regard to their potential to generate metastasis. However, it has been proposed that exosomes may shape the tumor microenvironment with different underlying mechanisms, depending on the exosome cargo [28]. Exosomes may be secreted within a tissue and can be found in the plasma, but they can also be released in many other biological fluids [29,30,31,32,33,34,35]. This means that the same approach of establishing differences in exosome counts between healthy individuals and those inflicted with a disease may be investigated using other body fluids.

There is also evidence that exosomes may actively contribute to the continuous genome remodeling during our lifetime. In fact, it has been shown that exosomes containing a reporter gene are released in vivo, circulating through blood and transferring the spermatozoa into the gonads, with a possible transfer of the acquired gene to the progeny [36].

The fact is that exosomes are considered a natural source of disease biomarkers [37,38,39,40,41,42,43,44,45,46,47,48,49,50,51,52,53,54,55,56,57,58,59,60,61,62,63]. The future goal of translational oncology is and will be to define the molecules’ cargo of body fluid-derived exosomes in tumor patients, also based on the evidence that tumor-released exosomes are involved in both tumor progression and metastasis [25,26,27,28,29,64]. However, to date, the data supporting the use of exosomes to identify new and valuable disease biomarkers have been below par. Several unexpected but interesting findings propose the simple measurement of exosome plasmatic levels as a key prognostic value [65,66,67]. The data also suggest that the increased exosome blood count is a hallmark of tumor patients as it is a common finding, regardless of tissue specificity [7,16,17,18,19].

## Conclusions

An intriguing paper has introduced a new term “Vesiclemia” [68], which means the presence of measurable plasmatic levels of extracellular vesicles in tumor patients. This paper adds further support to a new strategy in the follow-up of cancer patients that will take into careful account the plasmatic EV cargo, rather than the potential biomarkers’ cargo. However, the extracellular vesicle count appears to have potential significance in disease conditions other than tumors. In fact, recently, extracellular vesicle count has been proposed as a valuable new tool in infectious diseases [69]. Similar findings were reported in postmenopausal women taking hormonal replacement therapy [70]. Of course, the other disease conditions need clinical validation at least comparable to what we have to date for tumor patients. This editorial has emphasized a bulk of clinical results supporting the use of a “plasmatic exosome count” as a new valuable tool in the follow-up of tumor patients [7,17,18,19,20]. The plasmatic exosome count may be implemented by analyzing other components, including: (i) the exosome size (that has been proven to be smaller in tumor patients than in controls) [16,67]; (ii) the expression of known tumor markers [23,24,25] and (iii) the intraluminal pH of circulating exosomes [13].

Multicenter clinical studies are of course mandatory in order to validate the existing data in higher patient numbers. However, to perform longitudinal studies in patients undergoing either surgical or/and medical treatment is also mandatory [18], with the aim to use the plasmatic exosome count as a new tool in the clinical follow-up of cancer patients. Another important point is to extend the exosome count to other body fluids, with the purpose to limit unnecessary invasive procedures and reduce public health costs. The existing data on urine and other body fluids are very promising [29,30,31,32,33,34,35], but with limited data on the exosome count. A key series of data has shown that a major cause of the increased exosome release from tumors is the microenvironmental acidity [71]. Additionally, considering and deliberating on new anti-tumor therapies targeted to the tumor microenvironment rather than tumor cells [72] may lead to a reduced exosome release with a reduced risk of tumor metastasis [24,25,26,27].
